# Pregnancy and delivery outcomes in coronavirus disease 2019: a cross-sectional study from a tertiary center

**DOI:** 10.1590/1806-9282.20240842

**Published:** 2025-03-17

**Authors:** Suzan Şahin, Bülent Kaya, Emre Mat, Gazi Yıldız, Adil Barut

**Affiliations:** 1 Kartal Dr. Lütfi Kırdar City Hospital, Department of Infectious Diseases and Microbiology – İstanbul, Türkiye.; 2 Kartal Dr. Lütfi Kırdar City Hospital, Department of Obstetrics and Gynecology – İstanbul, Türkiye.; 3 İstanbul University-Cerrahpaşa, Faculty of Medicine, Department of Obstetrics and Gynecology – İstanbul, Türkiye.

**Keywords:** COVID-19, Pregnancy complications, Pregnancy outcome, SARS-CoV-2

## Abstract

**OBJECTIVE:**

The objective of this study was to document the clinical and laboratory characteristics and maternal and delivery outcomes of pregnant women with coronavirus disease 2019.

**METHODS:**

We retrospectively assessed pregnant women who had been admitted either with symptoms of coronavirus disease 2019 or for delivery from March 2020 to January 2023. The participants were evaluated in two cohorts: the initial cohort included all pregnant women hospitalized with coronavirus disease 2019, and the final cohort included those who delivered under the circumstances of coronavirus disease 2019.

**RESULTS:**

The initial cohort included 295 pregnant women with symptomless (n=124, 42%), mild (n=122, 41.4%), moderate (n=18, 6.1%), and severe (n=31, 10.5%) coronavirus disease 2019. Among comorbidities, hypothyroidism (n=40, 13.6%) had the highest share. The most frequent laboratory abnormality was elevated D-dimer levels (94.6%). The final cohort included 193 women. Eight patients (4.1%) required intensive care unit (ICU) stay. Mortality occurred in five patients (2.6%). Out of 121 cesarean section (SC) deliveries, 61.2% were on an emergency basis. Rates of preterm, low birth weight, and stillbirth were 18.7%, 14.5%, and 3.1%, respectively. Moderate-severe coronavirus disease 2019 showed significant inverse correlations with delivery week, birth weight, and APGAR scores at 1 and 5 min (p<0.0001).

**CONCLUSION:**

A considerable proportion of pregnant women with coronavirus disease 2019 experienced maternal and delivery complications, particularly preterm and low birth weight. Hypothyroidism had a notable share among comorbidities.

## INTRODUCTION

Pregnant women are vulnerable to respiratory pathogens and severe pneumonia due to their immunosuppressed condition and physiological adaptations during pregnancy, such as elevated diaphragm, increased oxygen consumption, and swelling of the respiratory tract mucosa, making them less tolerant to low oxygen levels^1–3^. These characteristics have been found to adversely affect pregnant women during previous outbreaks or pandemics^4,5^. The coronavirus disease 2019 (COVID-19) pandemic caused by severe acute respiratory syndrome coronavirus-2 (SARS-CoV-2) has affected pregnant women on a much greater scale when compared with previous pandemics.

After more than four years of fatal and devastating consequences, COVID-19 still remains on the agenda, irrespective of a presumably decreased level of severity risk of the latest variants. The World Health Organization (WHO), in its latest COVID-19 Epidemiological Update, emphasized the fact that "trends in the number of reported new cases and deaths should be interpreted with caution due to decreased testing and sequencing, alongside reporting delays in many countries. According to estimates obtained from wastewater surveillance, clinical detection of cases underestimates the real burden from 2- to 19-fold^6^." Therefore, COVID-19 in pregnant women continues to be under focus with respect to its clinical features and effects on maternal and delivery outcomes.

In this cross-sectional study, we aimed to document the clinical and laboratory characteristics and maternal and delivery outcomes of pregnant women with COVID-19. Our analysis included an initial cohort of all pregnant women hospitalized with COVID-19 and a final cohort of those who had deliveries at our center.

## METHODS

We retrieved hospital data on pregnant women (≥18 years of age) who had been admitted to the Obstetrics and Gynecology Clinic either with COVID-19 symptoms or for delivery and who were found to have COVID-19 on PCR testing between March 20, 2020, and January 1, 2023.

Data included sociodemographic characteristics, number of pregnancies, type of pregnancy, gestational week, comorbid conditions, vaccination status for COVID-19, type and time of delivery, the need for urgent cesarean section (CS), and neonatal outcomes. Acquired comorbidities (gestational hypertension, gestational diabetes, or preeclampsia) during pregnancy were excluded. Newborn PCR testing for COVID-19 was not performed.

Laboratory testing included whole blood count and biochemical parameters. Radiologic imaging was performed in patients with dyspnea only after the patient's informed consent.

### Ethical approval

The study protocol was approved by the institutional review board (25.01.2023-2022/514/242/15). All the procedures were conducted in compliance with the Declaration of Helsinki. Analysis and reporting of the results are in compliance with the Strengthening the Reporting of Observational Studies in Epidemiology (STROBE) checklist.

### Definitions

The severity of COVID-19 was classified based on symptom severity, as asymptomatic, mild, moderate, or severe^7^. Patients with no clinical symptoms suggestive of COVID-19 were classified as symptomless. Mild disease was characterized by the presence of flu-like symptoms (body temperature ≥38.3°C, cough, fatigue, headache, and/or myalgias), anosmia, and normal oxygenation (>97%) while the patient was breathing room air; moderate disease by, in addition to mild disease, dyspnea and oxygenation of at least 94%, and severe disease by, in addition to moderate disease, oxygenation of <94%.

Delivery outcomes were defined as abortus (<20 gestational weeks), preterm (20–36 weeks), and term (≥37 weeks) deliveries. Low birth weight was defined as a birth weight of less than 2.500 grams. Stillbirth was defined as the loss of a baby at or after 20 weeks of pregnancy.

Analyses were performed in two phases. The initial analysis included all pregnant women hospitalized during the pandemic and tested positive for COVID-19 (initial cohort), while the final analysis included those whose deliveries were performed during hospitalization (final cohort).

### Statistical analysis

Data were processed using the Statistical Package for Social Sciences (SPSS) version 21 (IBM Corp., Armonk, NY, USA). Quantitative data were expressed as means with standard deviation (SD), or medians with minimum-maximum or interquartile range (IQR), and qualitative data as frequencies and percentages. Homogeneity was checked using Levene's test where a p>0.05 was considered in favor of homogeneity. The Shapiro-Wilk normality test was used to check whether continuous variables were normally distributed. For pairwise comparisons, numerical variables were compared using the independent t-test if normally distributed. Multigroup comparisons of normally distributed variables were made using the one-way ANOVA test. The post-hoc multiple comparison (Bonferroni) test was used to determine between-group differences. Nominal variables were analyzed with Pearson's or Fisher's chi-squared test. Univariate and binary analyses were performed to determine the effect of COVID-19 severity on maternal and delivery outcomes. A p<0.05 was considered statistically significant. All variables were expressed with 95% confidence intervals (CIs).

## RESULTS

### The initial cohort

During the study period, 4,775 pregnant women were admitted to the Obstetrics and Gynecology Clinic and 295 met the eligibility criteria. The median age was 29 years (IQR 26–34).

Before admission, only 34 women (11.5%) had been vaccinated for COVID-19. The onset of symptoms ranged from 1 to 12 days. A total of 79 patients (26.8%) had comorbid conditions, for which they had been receiving ongoing treatments, including hypothyroidism, asthma, diabetes mellitus, hypertension, and others (Table 1). Hypothyroidism was the leading comorbid condition (n=40, 13.6%).

**Table 1:** Characteristics of pregnant women in the initial and final cohorts.

Characteristics		The initial cohort (n=295)	The final cohort (n=193)
Maternal age, median (IQR)		29 (26–34)	29 (25–34)
COVID-19 vaccinated		34 (11.5)	23 (11.9)
COVID-19 severity, n (%)			
	Symptomless	124 (42.0)	114 (59.1)
	Mild	122 (41.4)	66 (34.2)
	Moderate	18 (6.1)	1 (0.05)
	Severe	31 (10.5)	12 (6.2)
COVID-19 symptoms, n (%)			
	Cough	151 (51.2)	64 (33.2)
	Fever	46 (15.6)	16 (8.3)
	Shortness of breath	33 (11.2)	11 (5.7)
	Headache	55 (18.6)	20 (10.4)
	Myalgia	117 (39.7)	44 (22.8)
	Diarrhea	2 (0.7)	0
	Malaise	153 (51.9)	67 (34.7)
	Loss of sense of smell and/or taste	5 (1.7)	4 (2.07)
	Comorbidities, n (%)	79 (26.8)	50 (25.9)
	Hypothyroidism	40 (13.6)	25 (12.9)
	Asthma	15 (5.1)	8 (4.1)
	Diabetes mellitus	14 (4.7)	7 (3.6)
	Hypertension	9 (3.1)	7 (3.6)
Abnormal laboratory parameters, n (%)			
	Lymphopenia (lymphocyte count <1,000 mcL)	83 (28.1)	NA
	C-reactive protein (>5 mg/L)	242 (82.0)	NA
	Lactate dehydrogenase (>214 U/L)	230 (78.0)	NA
	D-dimer (>550 μg/L)	279 (94.6)	NA
	Fibrinogen (>400 mg/dL)	176 (59.7)	NA
	Ferritin (>150 μg/L)	45 (15.3)	NA
ICU admission, n (%)		11 (3.7)	8 (4.1)
Hospital stay (days), median (IQR)		4 (3–5)	3 (3–4)
Mortality, n (%)		NA	5 (2.6)

IQR: interquartile range; COVID-19: coronavirus disease 2019.

Out of 295 women, 124 (42%) were symptomless. The remaining patients had mild (n=122, 41.4%), moderate (n=18, 6.1%) or severe (n=31, 10.5%) COVID-19.

Radiologic imaging of the chest was performed in 42 patients. Computed tomography (CT) was performed in 33 patients, of whom 24 patients had ground glass opacities. Chest radiograms were obtained in nine patients, all showing signs of pulmonary involvement.

The median length of hospitalization was 4 days (IQR 3–5). Eleven patients required intensive care unit (ICU) admission due to worsening COVID-19, of whom three were discharged home after recovery.

### Abnormal laboratory parameters

The most frequent laboratory abnormality was an increased D-dimer level (94.6%), followed by an elevated C-reactive protein (CRP) level (82.0%) (Table 1). Three laboratory parameters showed significant differences across the severity groups. The highest incidence of lymphopenia (lymphocyte count <1.000 mcL) was seen with severe COVID-19 (p<0.0001). An abnormal CRP level (>5 mg/L) was most frequent in the severe group (100%), with a significantly higher incidence than that in the symptomless group (p=0.002) and similar to those in the mild and moderate groups. An abnormal ferritin level (>150 μg/L) was most frequent in the severe group (58.1%), being significantly higher when compared to the symptomless and mild groups (p<0.0001).

### Treatment

Throughout the pandemic, treatment was designed according to the recommendations of the Ministry of Health of Türkiye. A total of 24 women (8.1%) refused treatment. Patients whose oxygen saturation was <97% received supplemental oxygen and patients whose D-dimer levels were >550 μg/L received low-molecular-weight unfractionated heparin. During the early periods of COVID-19, patients received hydroxychloroquine (n=32) until May 2021 or lopinavir/ritonavir (n=26) until March 2021. Then, treatment was continued with prednisolone or dexamethasone based on the patient's clinical condition.

### The final cohort

Out of 295 patients, 193 women (65.4%) had deliveries during hospitalization (Table 1). Of these, 114 (59.1%) were symptomless, 66 (34.2%) had mild, and 13 (6.7%) had moderate-severe disease (1 moderate and 12 severe). The median age was 29 years (IQR 25–34). Only 23 (11.9%) had been vaccinated against COVID-19. Fifty patients (25.9%) had comorbidities, the most common being hypothyroidism in 25 patients (12.9%). Radiologically, CT was performed in 11 patients, showing ground glass opacities. Chest radiography of two patients showed bilateral diffuse pulmonary involvement.

### Delivery outcomes

Delivery outcomes of patients in the three severity groups are shown in Table 2. Deliveries included 121 CSs (62.7%) and 72 vaginal deliveries. A substantial proportion of CSs (61.2%, n=74) were performed on an emergency basis. There was no abortion. Thirty-six women (18.7%) had preterm deliveries. Twenty-eight women (14.5%) had low birth weight (<2,500 gr) newborns. A total of six stillbirths (3.1%) occurred.

**Table 2:** Comparison of variables based on the COVID-19 severity in the final cohort (n=193).

Parameters		Symptomless (n=114)	Mild (n=66)	Moderate-severe (n=13)	p
Age, years (mean±SD)		29.0±5.4	29.0±5.6	31.3±3.9	0.363
COVID-19 vaccinated, n (%)		19 (16.7)	4 (6.1)	0 (0.0)	**0.041**
Hypothyroidism		14 (12.3)	7 (10.6)	4 (33.3)	0.096
ICU admission		0	0	8 (61.5)	**0.0001**
Mortality, n (%)		0 (0.0)	0 (0.0)	5 (38.5)	**0.0001**
Delivery week, (mean±SD)		38.0±1.9	37.0±3.6	34.0±4.3	P_1_: 0.068 P_2_: **0.0001** P_3_: **0.002**
Type of delivery, n (%)					
	Normal vaginal delivery	44 (38.6)	27 (39.7)	1 (7.7)	0.134
	Emergency cesarean section, n (%)	40 (35.1)	23 (34.8)	11 (84.6)	**0.033**
Delivery outcomes, n (%)					
	Preterm	12 (10.5)	16 (24.2)	8 (61.5)	**0.0001**
	Term	102 (89.5)	50 (76.8)	5 (38.5)	
	Low birth weight	12 (10.5)	15 (23.1)	8 (61.5)	**0.001**
	Stillbirth	1 (0.9)	4 (6.1)	1 (7.7)	0.095
Birth weight (g), (mean±SD)		3,131±565	2,966±755	2,390±845	P_1_: 0.326 P_2_: **0.001** P_3_: **0.017**
APGAR 1 min (mean±SD)		7.8±0.6	7.4±1.9	5.5±3.3	P_1_: 0.161 P_2_ **: 0.0001** P_3_: **0.0001**
APGAR 5 min (mean±SD)		8.4±2.2	8.2±1.2	7.0±2.3	P_1_: 0.176 P_2_ **: 0.0001** P_3_: **0.009**

One-way ANOVA test (Bonferroni), SD: standard deviation; P_1_: symptomless-mild; P_2_: symptomless-moderate-severe; P_3_: mild-moderate-severe; CRP: C-reactive protein; LDH: lactate dehydrogenase; NS: not significant; COVID-19: coronavirus disease 2019; ICU: intensive care unit. Statistically significant values are indicated in bold

### Hospital outcomes

The median length of hospitalization was 3 (IQR 3–4) days. The median time from admission to delivery was 1 day (IQR 0–1). The longest hospital stay was 31 days for a patient who had also received ICU care for 19 days and had undergone emergency CS (low birth weight) before ICU admission.

### Intensive care unit admission

Eight (4.1%) patients with moderate-severe COVID-19 required ICU care for a median of 4 (IQR 2–14) days. Mortality due to COVID-19 occurred in five patients (2.6%), with none having been vaccinated.

### Results of statistical analysis

In the final cohort, patients with moderate-severe COVID-19 differed significantly from those in the symptomless and mild groups with the following characteristics: a lower vaccination rate, higher rates of ICU admission and mortality, earlier delivery week, higher rates of emergency CS, preterm birth, and low birth weight, as well as significantly decreased 1- and 5-min APGAR scores (Table 2). In the univariate analysis, moderate-severe disease showed significant inverse correlations with delivery week, birth weight, and APGAR 1- and 5-min scores (for all, p<0.0001). Mild disease showed significant inverse correlations only with delivery week (p=0.023) and APGAR 5-min score (p=0.015). In the binary analysis, moderate-severe disease was a highly independent predictor for preterm delivery, low birth weight, and emergency CS (Table 3).

**Table 3:** Results of binary analysis.

Variables		OR	95%CI	Z	p
Preterm delivery					
	Mild	2.6	1.1–5.9	2.21	**0.027**
	Moderate-severe	11.9	3.3–43.4	3.75	**0.0001**
Low birth weight					
	Mild	2.6	1.11–5.85	2.21	**0.027**
	Moderate-severe	11.9	3.26–43.4	3.75	**0.0001**
Emergency CS					
	Mild	1.1	0.50–2.5	0.267	0.789
	Moderate-severe	8.5	1.0–69.6	2.001	**0.045**

Refence, symptomless group. CS: cesarean section; CI: confidence interval; OR: odds ratio. Statistically significant values are indicated in bold.

## DISCUSSION

### Severity of coronavirus disease 2019

In parallel with previous reports on pregnant women with COVID-19^8–10^, the great majority of the patients in the initial cohort were symptomless (42%) or had mild disease (41.3%). The incidence of ICU admission was low, being 3.7 and 4.1% in the initial and final cohorts, respectively. Despite the presence of higher rates (40%)^11^, several studies reported ICU admission rates similar to that found in the current study^12–14^.

A study from Türkiye investigated associations of two clinical scoring systems that involved formulas (neutrophil count multiplied by platelet or monocyte count, divided by the lymphocyte count). Scores of these indices were significantly higher in complicated cases with COVID-19^15^. In support of this finding, we consistently found the highest rates of lymphopenia in patients with severe COVID-19.

In the final cohort, relatively high rates of unfavorable outcomes were observed, particularly for preterm deliveries (18.7%), low birth weight newborns (14.5%), and stillbirths (3.1%). In addition, emergency CS accounted for 61.2% of all CSs. Cesarian section was also reported to be the most common indication arising from worsening maternal status^16^. Libretti et al.^17^ reported the rates of emergency CS, preterm deliveries, and low birth weight as 49%, 8.3%, and 8.3%, respectively, with no incidence of stillbirths. Cojocaru et al.^18^ reported preterm deliveries in 10.6% and stillbirths in 1.6% of the participants.

### Comorbid conditions

More than a quarter of women in the initial (26.8%) and final (25.9) cohorts had at least one comorbidity. The most common was hypothyroidism, with corresponding figures of 13.6 and 12.9%. In a study assessing patients with severe-critical COVID-19, the most common comorbid condition was immune-related diseases (5.8%), followed by asthma (5.2%), which was also the leading health problem (14.7%) among the non-survivors^16^.

There have been several reports showing that hypothyroidism does not per se constitute a risk factor for contracting COVID-19^19^ or affecting the course of COVID-19 severity^20^. However, the adverse effects of hypothyroidism on obstetric and neonatal outcomes have somewhat been well established^21^. In the present study, the potential adverse effects of hypothyroidism on obstetric and neonatal outcomes were seen in all COVID-19-related outcome parameters. First, the incidence of hypothyroidism was considerably higher at 33.3% among patients with moderate-severe COVID-19 as compared with 10.6% among those with mild disease and 12.3% among those with a symptomless course. Second, none of the eight patients requiring ICU admission had any comorbidity other than hypothyroidism, which was the case in two patients (25%). Third, mortality due to severe COVID-19 occurred in five patients, of whom two (40%) had hypothyroidism, but no other comorbidity. Fourth, hypothyroidism was the most frequent comorbid condition among patients who required emergency CS, accounting for 12 cases (16.2%), followed by asthma (8.1%), diabetes mellitus (8.1%), and hypertension (5.4%). Finally, hypothyroidism also showed strikingly high frequencies in relation to delivery outcomes, i.e., for preterm deliveries, of 13 patients with comorbidities, six (46.1%) had hypothyroidism; for deliveries resulting in low birth weight newborns, of six patients with comorbidities, three (50%) had hypothyroidism, and for six deliveries resulting in stillbirths, hypothyroidism was the only comorbidity in one patient. These findings may warrant further comparative studies about the possible adverse role of hypothyroidism in pregnant women with COVID-19.

### Limitations

The present study has three main limitation:s its retrospective design, the longer duration included in the analysis throughout diverse waves of the COVID-19 pandemic, and the lack of a comparison group.

## CONCLUSION

As compared with previous reports, pregnancy and delivery outcomes of women with COVID-19 were found to be poorer with respect to emergency CS, preterm deliveries, low birth weight newborns, and stillbirths, all of which showed a notable accompaniment with hypothyroidism.
